# The Antioxidant Alpha-Lipoic Acid Inhibits Proliferation and Invasion of Human Gastric Cancer Cells via Suppression of STAT3-Mediated MUC4 Gene Expression

**DOI:** 10.1155/2019/3643715

**Published:** 2019-12-13

**Authors:** Yu Yang, Erhu Fang, Jiajun Luo, Hongxue Wu, Yue Jiang, Ying Liu, Shilun Tong, Zhihua Wang, Rui Zhou, Qiang Tong

**Affiliations:** ^1^Department of Gastrointestinal Surgery I Section, Renmin Hospital of Wuhan University, Wuhan 430060, China; ^2^Department of Pediatric Surgery, Tongji Hospital, Tongji Medical College, Huazhong University of Science and Technology, Wuhan 430030, China; ^3^Central Laboratory, Renmin Hospital of Wuhan University, Wuhan 430060, China; ^4^Hubei Province Key Laboratory of Allergy and Immunology, School of Basic Medical Sciences, Wuhan University, Wuhan 430071, China

## Abstract

**Background:**

Metastasis and invasion are the main causes of mortality in gastric cancer. To improve the treatment of gastric cancer, the development of effective and innovative antitumor agents toward invasion and proliferation is needed. Alpha-lipoic acid (ALA), a naturally occurring thiol antioxidant, showed antiproliferative and cytotoxic effects on several cancers. So it is feasible to explore whether ALA can be used to inhibit proliferation and invasion in human gastric cancer.

**Methods:**

The expression of MUC4 in human gastric cancer tissues was assayed by immunohistochemistry. Then, we performed *in vitro* cell proliferation and invasion analysis to explore the antitumor effect of ALA using AGS, BGC-823, and MKN-28 cells. To further explore the mechanism of ALA-mediated downregulation of MUC4, we cotransfected human gastric cancer cells with STAT3 siRNA and STAT3 overexpression construct. ChIP assays were carried out to find the relationship between MUC4 and STAT3.

**Results:**

We found that the MUC4 gene was strongly expressed in human gastric cancer tissues. Meanwhile, ALA reduced proliferation and invasion of human gastric cancer cells by suppressing MUC4 expression. We also found that STAT3 was involved in the inhibition of MUC4 by ALA. Mechanistically, ALA suppressed MUC4 expression by inhibiting STAT3 binding to the MUC4 promoter region.

**Conclusion:**

ALA inhibits both proliferation and invasion of gastric cancer cells by suppression of STAT3-mediated MUC4 gene expression.

## 1. Introduction

Gastric cancer is the fifth most common cancer throughout the world, and it is the third leading cause of mortality related to cancer [1]. Most gastric cancer patients have had adjacent organs or distant metastasis, which is the main cause of death in gastric cancer patients. Although there has been great progress in gastric cancer treatment in the clinic, the outcomes of gastric cancer patients are still not satisfied [2]. Thus, it is necessary to find effective and innovative antitumor agents which can inhibit proliferation and invasion of gastric cancer.

The stability of redox plays a vital role in the normal growth of cells. However, there is continuous and abundant production of reactive oxygen species (ROS) in tumor cells, which promote tumor growth by causing DNA damage and reprogramming cell metabolism [3]. The overproduction of ROS without proper management is called oxidative stress. Alpha-lipoic acid (ALA) is a coenzyme of pyruvate dehydrogenase and glycine decarboxylase synthesized in mitochondria [4]. As a powerful antioxidant, ALA can not only clear the excessive ROS directly but also regenerate endogenous antioxidants such as vitamin C, vitamin E, coenzyme Q10, glutathione, and ALA itself [5]. ALA affects the process of free radical scavenging in cells, such as increasing glutathione synthesis and regulating activity of transcription factors [6]. Nowadays, ALA is widely used in the clinical treatment of diseases associated with excessive oxidative stress, such as diabetic peripheral neuropathy [7]. In recent years, ALA has been used as an anticancer agent in experimental studies of different cancers and achieved satisfying results [8, 9]. However, the underlying molecular mechanism is still unclear.

Mucins are high-molecular-weight glycoproteins, which can maintain integrity and lubricate and protect surfaces of epithelia [10]. To date, at least eighteen different mucin genes have been identified [11]. Mucin 4 (MUC4) is membrane-bound mucin, which is expressed in normal gastric mucosa and gastric cancer [12]. Recent research demonstrated that MUC4 is involved in the oncogenesis, differentiation, proliferation, invasion, and migration of tumors and can be used as a reference indicator for the evaluation of some tumor conditions. It has been reported that activator protein- (AP-) 2*α* inhibits MUC4 expression which in turn suppresses proliferation and invasion of pancreatic cancer cells [13]. Besides, the expression of MUC4 is mediated through upregulation of signal transducer and activator of transcription (STAT) in pancreatic cancer and gastric cancer [10, 14].

The current study was carried out to identify the effects of ALA on human gastric cancer progression. We found that MUC4 was upregulated in gastric cancer compared to normal tissues. ALA decreased STAT3 binding to MUC4 promoter region, repressed MUC4 expression, and consequently inhibited proliferation and invasion of human gastric cancer cells. Our data provide an in-depth mechanism by which ALA inhibits proliferation and invasion of gastric cancer cells, which validates the clinical use of ALA as a potential agent to enhance treatment outcomes in gastric cancer patients.

## 2. Materials and Methods

### 2.1. Patients and Samples

A total of 240 patients were diagnosed with gastric adenocarcinoma and underwent radical gastrectomy at Renmin Hospital of Wuhan University from June 2014 to July 2015. None of them received either preoperative chemotherapy or radiotherapy. Preoperative written consent was obtained from each patient. Primary lesion and corresponding noncancerous tissues were kept during operation and then were embedded in paraffin for immunohistochemistry. The depth of invasion was observed by the surgeon during the operation. Lymph node metastasis was observed by pathological examination. Distant metastasis was confirmed according to imageology such as computed tomography and positron emission tomography. All patients were followed up until August 2018, with a total of 12 cases (5% patients) lost in follow-up period. This study was approved by the Ethics Committee of Renmin Hospital of Wuhan University.

### 2.2. Cell Culture and Reagents

Human gastric cancer cell lines are as follows: AGS, BGC-823, and MKN-28 cells. AGS cell line was purchased from American Type Culture Collection (ATCC, Manassas, USA); BGC-823 and MKN-28 cell lines were gifts from Tongji Medical College, Huazhong University of Science and Technology (Wuhan, China). The cells were cultured in Roswell Park Memorial Institute- (RPMI-) 1640 medium supplemented with 10% fetal bovine serum (FBS). Cells were maintained in a 37°C incubator with 5% CO_2_. All cell lines tested negative for mycoplasma. Alpha-lipoic acid and TNF-*α* were purchased from Solarbio (Beijing, China). Anti-MUC4 antibody, anti-STAT3 antibody, and specific primary antibody were purchased from Sigma-Aldrich.

### 2.3. Immunohistochemistry

Paraffin-embedded tissue samples were cut into 4 *μ*m thick sections and mounted on poly-L-lysine-coated slides. Samples were dewaxed in xylene and rehydrated using a graded series of ethanol solutions. After deparaffinization, endogenous peroxidase activity was blocked by incubation in a 3% peroxide-methanol solution at room temperature (RT) for 10 min, and then, antigen retrieval was performed at 100°C in an autoclave for 7 min. Samples were then incubated at RT for 30 min. Afterward, sections were washed with phosphate-buffered saline (PBS) 3 times, 5 min each time. They were then incubated with rabbit anti-MUC4 antibody (Sigma-Aldrich, USA). Thoroughly washing with PBS was then performed, and primary antibody binding was visualized under a microscope.

### 2.4. Cell Transfection

Cells were plated in 6-well plates with RPMI-1640 medium supplemented with 10% medium FBS for 24 h before transfection. Transfections were performed using siRNAs and Lipofectamine RNAiMAX (Thermo Fisher) transfection reagent diluted in RPMI-1640 medium. Indicated plasmids were transfected using Lipofectamine 2000 (Thermo Fisher) according to the manufacturer's instruction.

### 2.5. Quantitative Real-Time PCR (qRT-PCR)

The total RNA was extracted from tissues using TRIzol Reagent (Thermo Fisher, USA) according to the manufacturer's instructions. Then, RNA was reverse transcribed to cDNA with 1 *μ*g total RNA, using reverse transcriptase and Oligo dT primers (Takara, Japan). The cDNA was then amplified with specific primers by PCR. The primers used for PCR were listed below. The conditions for qRT-PCR were as follows: 95°C for 3 min, followed by 40 cycles of 10 s at 95°C,10 s at 60°C, and 15 s at 70°C, followed by heating from 65°C to 95°C. Primers for qRT-PCR are listed as follows: MUC4 forward primer 5′-CTTCAGATGCGATGGCTACA-3′ and reverse primer 5′-GTTTCATGCTCAGGTGCTCA-3′, STAT3 forward primer 5′-GGCCATCTTGAGCACTAAGC-3′ and reverse primer 5′-CGGACTGGATCTGGGTCTTA-3′, 18S rRNA forward primer 5′-CGGCTACATCCAAGGAA-3′ and reverse primer 5′-GCTGGAATTACCGCGGCT-3′.

### 2.6. Western Blotting

Total protein was extracted from cells and its concentration was measured by BCA Protein Assay Kit (Solarbio, China). The protein samples were separated by SDS-PAGE and transferred to polyvinylidene fluoride (PVDF) membranes. After blocking, the membranes were incubated with specific primary antibodies overnight at 4°C and secondary antibody for 1 h at room temperature. Protein expression levels were normalized to *β*-actin. Densitometric scanning (Bio-Rad) was used to determine relative protein band intensity.

### 2.7. MTT

Cells were digested into a single cell suspension and were seeded into a 96-well plate at 5000 cells per well in 200 *μ*L. After coincubating with indicated concentration of ALA for 24 h, MTT solution (5 mg/mL, prepared with PBS, pH = 7.4, Solarbio, China) was added into the medium at 10 *μ*L per well. Cells were incubated for another 4 h, then the culture was terminated, and the supernatant was carefully absorbed and discarded. 100 *μ*L DMSO was added to each well and oscillated for 10 min to fully melt the crystallites. The wavelength of 490 nm was selected to determine the absorbance of each well.

### 2.8. Matrigel Invasion Assay

The cell invasion assay was performed using the BioCoat™ Matrigel apparatus (Corning Inc., USA), with RPMI-1640 medium supplemented with 10% medium FBS as the chemoattractant in the lower chamber. AGS cells (10^5^) in 300 *μ*L were added to the upper chamber, with or without addition of ALA or anti-MUC4, to invade the Matrigel for 24 h. Noninvading cells on the upper surface were removed, and invading cells on the lower surface were stained with the Diff-Quick stain kit (Solarbio, China). The number of invasion cells was counted by a phase contrast microscope.

### 2.9. Chromatin Immunoprecipitation

The chromatin immunoprecipitation assay (ChIP) kit was purchased from Abcam, United Kingdom. Briefly, AGS (6.0 × 10^6^) cells were fixed with 1% of formaldehyde. Genomic DNA was sheared to lengths ranging from 200 to 1000 bp with a Sonic Dismembrator (Fisher Scientific): Ampl 80%, 3 seconds on, 10 seconds off, for 10 cycles. One percent of the cell extract was taken as “input,” and the rest of the extract was incubated with anti-STAT3 or control IgG overnight at 4°C, followed by precipitation with protein A agarose beads. The immunoprecipitates were sequentially washed with a low salt buffer, a high salt buffer, a LiCl buffer, and with TE buffer. The DNA-protein complex was eluted and proteins were then digested with proteinase K. The DNA was detected by qRT-PCR analysis, and the data obtained by qRT-PCR for specific antibody were normalized to IgG control and plotted as percent input. qRT-PCR was performed using two different sets of primers: Primer set 1: 5′-TCATAC AGCCCCAAGGTCGC-3′ (sense) and 5′-TAGCCGGGTTCCTGGGTCC-3′ (antisense), corresponding to the MUC4 promoter region 3251–3373 (NCBI sequence, accession number AF241535), and Primer set 2: 5′-GAAAAGGGTGATTAGCGTGG-3′ (sense) and 5′-TCCCCTCAGGCGGCTGGCC-3′ (antisense), corresponding to the 3528–3632 region of MUC4 promoter.

### 2.10. Statistical Analysis

Statistical differences were determined by two-tailed *t*-test in two-group comparisons. The correlation between MUC4 and tumor clinicopathologic characteristics was analyzed by the chi-square test. *p* < 0.05 was considered statistically significant. IBM SPSS Statistics version 21.0 and GraphPad Prism version 7.0 were used to analyze data.

## 3. Results

### 3.1. MUC4 Gene Was Strongly Expressed in Human Gastric Cancer Tissues

**Figure 1:**
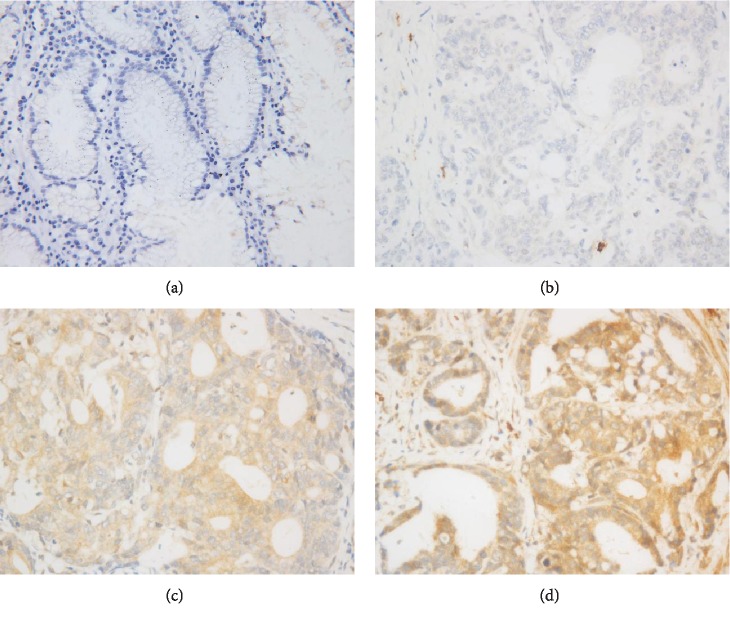
Expression of MUC4 in human gastric tissues. (a) Negative expression of MUC4 in normal gastric samples located away from cancer (400x). (b–d) Low, modest, and high expression of MUC4 in gastric cancer cells, respectively (400x).

To identify the expression of MUC4 in human gastric cancer and normal gastric tissues, we performed immunohistochemistry staining on normal gastric samples located away from cancer (*n* = 240) and gastric cancer (*n* = 240), respectively. As shown in Figure 1, the expression of MUC4 in normal gastric samples was negative (Figure 1(a)), and the expression of MUC4 in gastric cancer was positive by contrast (Figures 1(b)–1(d)). As shown in Table 1, the expression of MUC4 showed positive in 184 out of 240 gastric cancer tissues, while the expression of MUC4 showed positive in 80 out of 240 normal gastric tissues. These results thus demonstrated that MUC4 is significantly upregulated in human gastric cancer tissues compared with normal gastric tissues (Table 1, *p* < 0.001). Additionally, chi-squared tests demonstrated significant correlations between the expression of MUC4 and age, depth of invasion, TNM stage, lymph node metastasis, and distant metastasis (Table 2).

### 3.2. Alpha-Lipoic Acid Significantly Inhibits Endogenous MUC4 Expression in Human Gastric Cancer Cells

**Figure 2:**
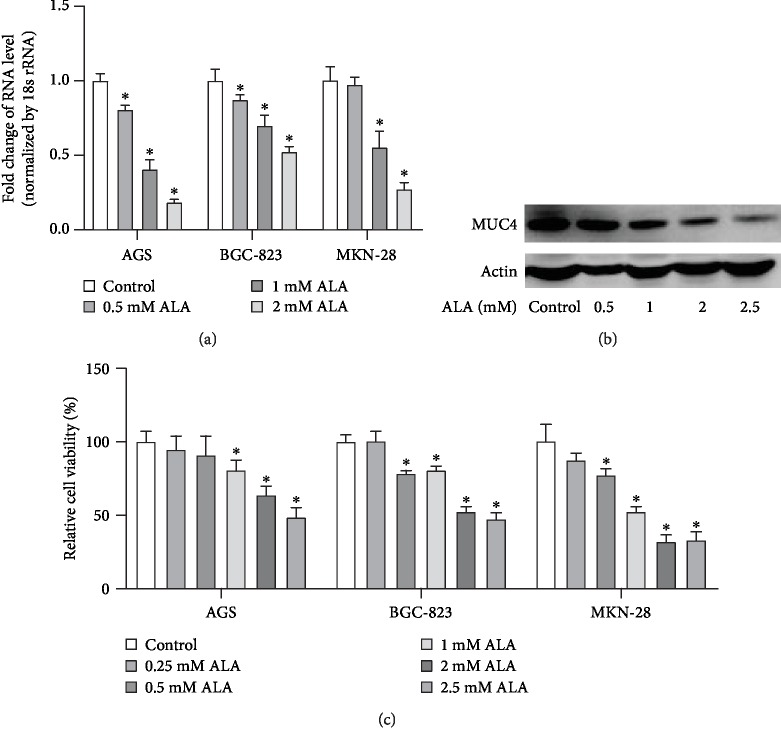
Alpha-lipoic acid inhibits the expression of endogenous MUC4 in human gastric cancer cells. (a) When cell confluence was 60%, AGS, BGC-823, and MKN-28 cells were incubated with 0, 0.5, 1, or 2 mM ALA for 24 h in RPMI with 10% FBS. After incubation, MUC4 mRNA in the cell lysates was examined by RT-PCR. (b) AGS cells were incubated with 0, 0.5, 1, 2, or 2.5 mM ALA for 24 h in RPMI with 10% FBS. Then, MUC4 protein was tested by western blotting. (c) AGS, BGC-823, and MKN-28 cells were incubated with 0, 0.25, 0.5, 1, 2, or 2.5 mM ALA for 24 h, and then, the viability was tested by the MTT method. Data represent mean ± SD from 3 independent experiments. ^∗^*p* < 0.05 compared to the control group.

To identify the effect of ALA on the endogenous expression of MUC4 in human gastric cancer cells, we incubated AGS, BGC-823, and MKN-28 cells with 0, 0.5, 1, or 2 mM ALA for 24 h in RPMI with 10% FBS when cell confluence was 60%. MUC4 mRNA in the cell lysates was examined by RT-PCR. As shown in Figure 2(a), ALA significantly inhibited the endogenous MUC4 expression in gastric cancer cells in a dose-dependent manner. Complementarily, AGS cells were incubated with 0, 0.5, 1, 2, or 2.5 mM ALA for 24 h in RPMI with 10% FBS, then MUC4 protein was measured by western blotting. As shown in Figure 2(b), ALA significantly inhibited the expression of endogenous MUC4 in AGS cells. We next sought to examine the effect of ALA on the viability of human gastric cancer cells; for this, AGS, BGC-823, and MKN-28 cells were incubated with 0, 0.25, 0.5, 1, 2, or 2.5 mM ALA for 24 h followed by MTT assay. As shown in Figure 2(c), significant decreases of cancer cell viability were detected in cells treated with 1, 2, and 2.5 mM ALA.

### 3.3. Alpha-Lipoic Acid Inhibits TNF-*α*-Induced MUC4 in Gastric Cancer Cells

**Figure 3:**
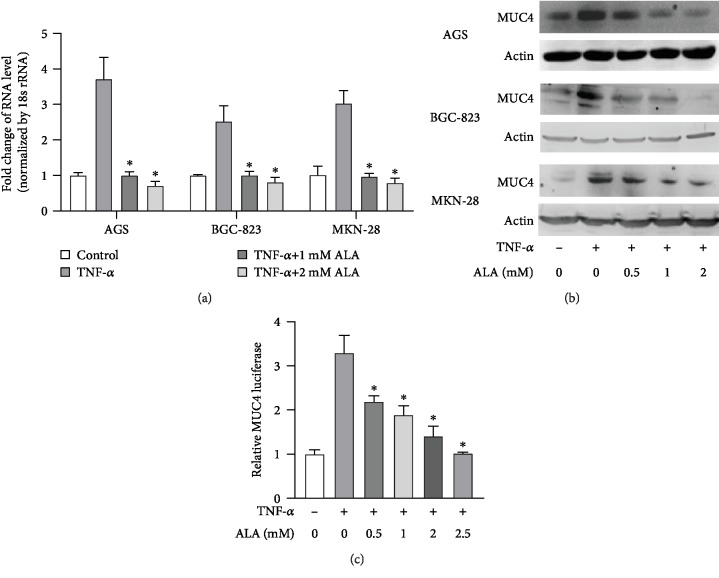
Alpha-lipoic acid inhibits TNF-*α*-induced MUC4 in gastric cancer cell lines. (a) AGS, BGC-823, and MKN-28 cells pretreated with the indicated concentrations of ALA for 24 h were treated with 20 ng/mL TNF-*α* for 4 h. After TNF-*α* treatment, RT-PCR was performed to analyze the MUC4 mRNA. (b) AGS, BGC-823, and MKN-28 cells pretreated with 0, 0.5, 1, or 2 mM ALA for 24 h in RPMI with 10% FBS were treated with or without 20 ng/mL TNF-*α*. And then, western blotting was performed to analyze the expression of MUC4 at protein levels. (c) The pGL3-MUC4 promoter construct-transfected AGS cells were pretreated with the indicated concentrations of ALA for 24 h, then incubated with 20 ng/mL TNF-*α* for 4 h and luciferase activity was determined using a luminometer. Data represent mean ± SD from 3 independent experiments. ^∗^*p* < 0.05 compared to only TNF-*α* treatment group.

We sought to determine whether ALA can suppress TNF-*α*-induced MUC4 in human gastric cancer; to this end, we pretreated AGS, BGC-823, and MKN-28 cells with the indicated concentrations of ALA for 24 h followed by exposure of the cells with 20 ng/mL TNF-*α* for 4 h. After TNF-*α* treatment, RT-PCR was performed to analyze the MUC4 mRNA. As shown in Figure 3(a), TNF-*α* significantly induced MUC4 expression, and pretreatment of ALA significantly inhibited TNF-*α*-induced expression of MUC4 in gastric cancer cells. Complementarily, AGS, BGC-823, and MKN-28 cells pretreated with 0, 0.5, 1, or 2 mM ALA for 24 h in RPMI with 10% FBS were treated with or without 20 ng/mL TNF-*α*. And then, western blotting showed that the expression of MUC4 was significantly decreased in the presence of ALA (Figure 3(b)). In addition, the pGL3-MUC4 promoter construct-transfected AGS cells were pretreated with the indicated concentrations of ALA for 24 h, then incubated with 20 ng/mL TNF-*α* for 4 h. Luciferase activity was determined using a luminometer. As shown in Figure 3(c), the relative MUC4 luciferase activity was significantly decreased in cells treated with ALA at higher concentrations. Taken together, ALA inhibits TNF-*α*-induced MUC4 in gastric cancer cells.

### 3.4. Alpha-Lipoic Acid Inhibits AGS Cell Invasion by Suppressing MUC4 Expression

**Figure 4:**
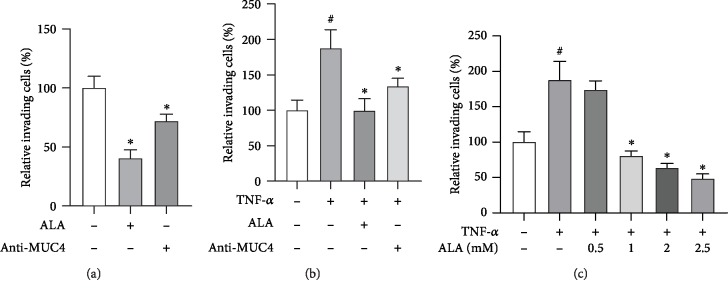
Alpha-lipoic acid inhibits invasion of AGS cells by suppressing MUC4 expression. (a) AGS cells were incubated with either 2 mM ALA or 200 ng/mL MUC4 antibody in a BioCoat™ Matrigel apparatus with 8 *μ*M pore membrane for 24 h. (b) AGS cells were incubated with 20 ng/mL TNF-*α* in the presence or absence of 2 mM ALA or 200 ng/mL MUC4 antibody in a BioCoat™ Matrigel apparatus for 24 h. (c) AGS cells were incubated with 20 ng/mL TNF-*α* in the presence of 0-2.5 mM alpha-lipoic acid or 200 ng/mL MUC4 antibody. After incubation, the cells that invaded the lower surface of the chambers were counted using a phase contrast light microscope after staining with a Diff-Quick Stain kit. Data represent the mean ± SD from 3 independent experiments. ^#^*p* < 0.05 versus control; ^∗^*p* < 0.05 versus TNF-*α* only.

We then tested whether ALA suppressed invasion of the gastric cancer cells. Matrigel invasion assay showed that the number of relative invading cells was significantly decreased in the ALA-treated group compared with the control group and anti-MUC4 showed a similar effect (Figure 4(a)). Interestingly, the effect can also be observed after TNF-*α* treatment (Figure 4(b)). In addition, the higher the concentration of ALA, the fewer number of relative invading cells was observed (Figure 4(c)).

### 3.5. STAT3 Is Involved in the Inhibition of MUC4 by Alpha-Lipoic Acid

**Figure 5:**
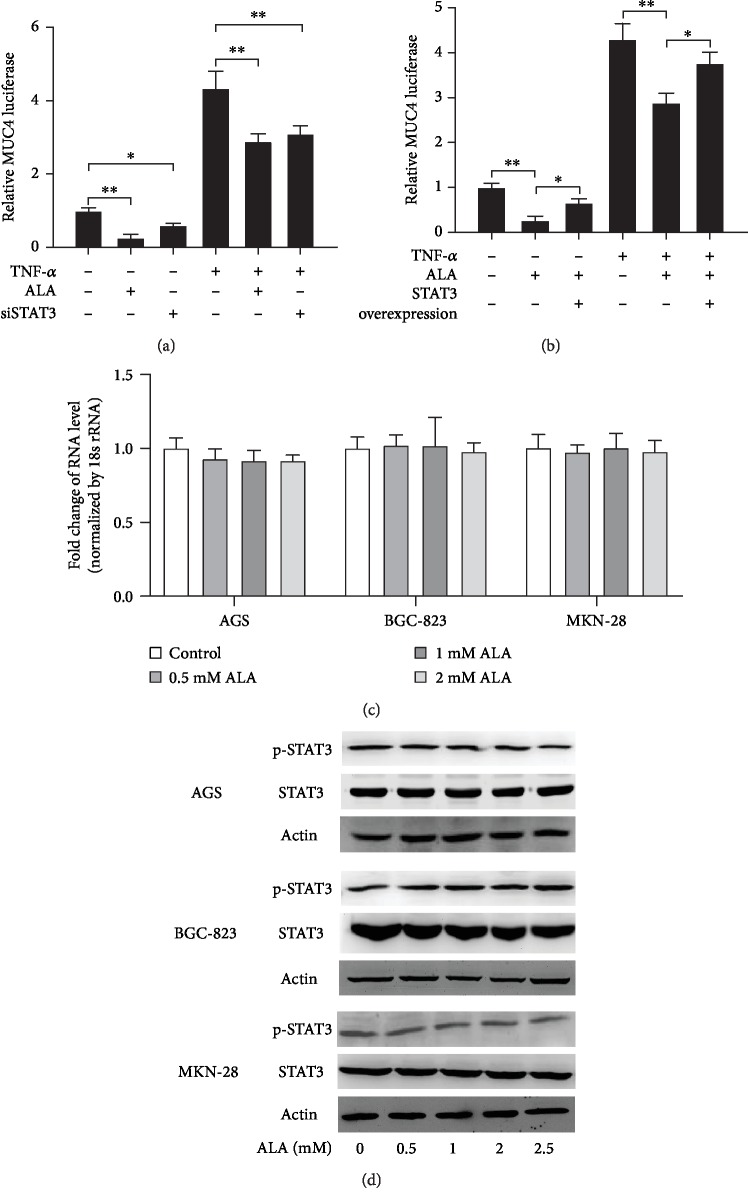
The role of STAT3 in the inhibition of MUC4 by alpha-lipoic acid in gastric cancer cells. (a) The STAT3 siRNA was cotransfected with pGL3-MUC4 promoter construct into AGS cells pretreated with 2 mM ALA. After incubation with 20 ng/mL TNF-*α* for 4 h, luciferase activity was determined using a luminometer. (b) STAT3 overexpression construct was cotransfected with a pGL3-MUC4 promoter construct into AGS cells. The transfected cells pretreated with or without 2 mM ALA were incubated with 20 ng/mL TNF-*α* for 4 h, and then, MUC4 luciferase activity was determined using a luminometer. (c) When cell confluence was 60%, AGS, BGC-823, and MKN-28 cells were incubated with 0, 0.5, 1, or 2 mM ALA for 24 h in RPMI with 10% FBS. After incubation, STAT3 mRNA in the cell lysates was examined by RT-PCR. (d) AGS, BGC-823, and MKN-28 cells pretreated with 0, 0.5, 1, 2, or 2.5 mM ALA for 24 h in RPMI with 10% FBS were treated with or without 20 ng/mL TNF-*α*. And then, western blotting was performed to analyze p-STAT3 and STAT3 protein level. Data represent the mean ± SD from 3 independent experiments.

It has been reported that STAT3 plays a role in human gastric cancer development. To identify whether STAT3 is involved in inhibition of MUC4 by ALA in gastric cancer cells, the STAT3 siRNA was cotransfected with pGL3-MUC4 promoter construct into AGS cells pretreated with 2 mM ALA. After incubation with 20 ng/mL TNF-*α* for 4 h, the luciferase was determined using a luminometer. The relative MUC4 luciferase activity was significantly decreased in STAT3-transfected AGS cells compared to the control group (Figure 5(a)). Complementarily, STAT3 overexpression construct was cotransfected with a pGL3-MUC4 promoter construct into AGS cells. The transfected cells pretreated with or without 2 mM ALA were incubated with 20 ng/mL TNF-*α* for 4 h, and then, the MUC4 luciferase activity was determined using a luminometer. As shown in Figure 5(b), the relative MUC4 luciferase activity was significantly higher in STAT3 overexpression cells than in cells only incubated with ALA. These two assays indicated that the expression of MUC4 gene is regulated by STAT3. Furthermore, to explore the role of STAT3 in the inhibition of MUC4 by ALA in gastric cancer cells, AGS, BGC-823, and MKN-28 cells were incubated with 0, 0.5, 1, or 2 mM ALA for 24 h in RPMI with 10% FBS when cell confluence was 60%. After incubation, STAT3 mRNA in the cell lysates was examined by RT-PCR. There was no significant difference between the STAT3 mRNA levels in cells incubated with indicated concentrations of ALA (Figure 5(c)). In addition, AGS, BGC-823, and MKN-28 cells pretreated with 0, 0.5, 1, 2, or 2.5 mM ALA for 24 h in RPMI with 10% FBS were treated with or without 20 ng/mL TNF-*α*. And then, western blotting was performed to analyze p-STAT3 and STAT3 protein levels. There was no significant difference between the p-STAT3 and STAT3 protein levels of cells incubated with indicated concentrations of alpha-lipoic acid. Taken together, STAT3 was involved in the inhibition of MUC4 by ALA, but its expression level was not affected.

### 3.6. Alpha-Lipoic Acid Inhibits STAT3 Binding to the MUC4 Promoter Region

**Figure 6:**
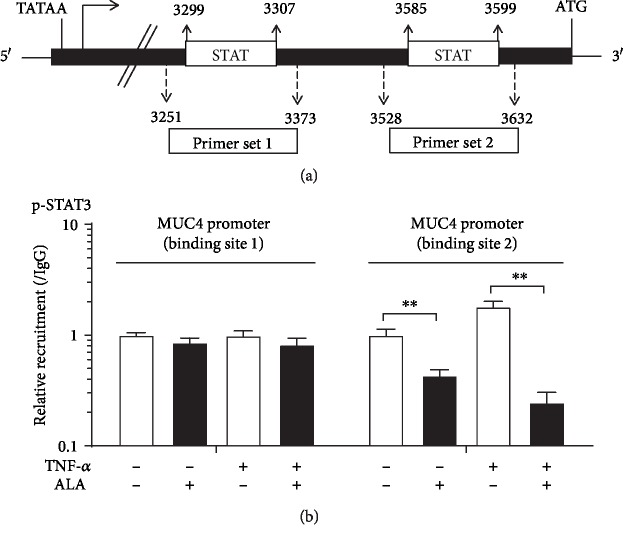
Alpha-lipoic acid inhibits STAT3 binding to the MUC4 promoter region. (a) Schematic representation of MUC4 promoter. The two putative STAT-binding sites as well as the primers used for chromatin immunoprecipitation experiments (Primer set 1 and Primer set 2) are shown. (b) Binding of p-STAT3 to MUC4 promoter by ALA with or without TNF-*α* treatment. AGS cells were treated with 2 mM ALA overnight and then exposed to 20 ng/mL TNF-*α* for 4 h, followed by ChIP assay using anti-p-STAT3 and PCR primers covering 2 different regions of the MUC4 promoter. Data represent the mean ± SD from 3 independent experiments. ^∗∗^*p* < 0.05.

Figure 6(a) shows the schematic representation of MUC4 promoter. The two putative STAT-binding sites as well as the primers used for chromatin immunoprecipitation experiments (Primer set 1 and Primer set 2) are shown. AGS cells were treated with 2 mM ALA overnight and then exposed to 20 ng/mL TNF-*α* for 4 h or without TNF-*α* treatment, followed by ChIP assay using anti-p-STAT3 and PCR primers covering 2 different regions of the MUC4 promoter. As shown in Figure 6(b), ALA inhibits STAT3 binding to site 2 in the MUC4 promoter region.

## 4. Discussion

In the present study, we demonstrated the inhibitory effect of ALA on human gastric cancer cell proliferation and invasion. The possible mechanism of this inhibitory effect was through suppression of MUC4. ALA inhibited STAT3 binding to the MUC4 promoter region, reduced the expression of MUC4, which in turn inhibited the proliferation and invasion of gastric cancer cells. To our knowledge, this is the first study showing the effect of ALA on resistance to cell proliferation and invasion in gastric cancer.

MUC4 protects cancer cells during hematological transmission and promotes the invasion and colonization of cancer cells to metastatic sites [15]. Many studies have shown that high MUC4 expression in the gastric cancer is related to the poor prognosis, such as lymph node metastasis and vascular invasion, which is the main cause of death in patients with gastric cancer [16]. We performed immunohistochemistry staining, the results showed positive MUC4 expression in gastric cancer tissues compared with normal tissues (Figure 1 and Table 1, *p* < 0.001), which is in accordance with known study results. Immunohistochemistry of prognostic factors showed that there were significant differences in MUC4 expression under different conditions such as ages, depth of invasion, TNM stage, lymph node metastasis, and distant metastasis (Table.  2, *p* < 0.05). MUC4 has been further studied as a target for treating various types of cancer, such as breast cancer [17] and colorectal cancer [18]. Since MUC4 was closely related to gastric cancer, it may be possible to suppress gastric cancer by regulating MUC4 expression.

It has been reported that ALA has a protective effect on gastric ulcer in rats because of its antioxidant and anti-inflammatory properties [19]. In view of the inhibitory effect of ALA on the proliferation and metastasis in various cancer cells [6], we treated gastric cancer cells with ALA and found that 1 mM ALA can reduce the viability of gastric cancer cells (Figure 2(c)). Its inhibitory effect on gastric cancer cells may be related to the inhibition of MUC4 expression (Figures 2(a) and 2(b)).

TNF-*α* and IL-6 can both induce MUC4 gene expression. To explore the mechanism, we found that the effect of ALA on gastric cancer cells might be related to STAT3. Therefore, we hope that the drugs or factors can induce exogenous MUC4 without affecting the expression level of STAT3. However, it has been reported that IL-6 may activate STAT3, leading to transcriptional upregulation of downstream growth-related genes [20]. On the contrary, TNF-*α* induces MUC4 through independent STAT3 pathway (such as NF-*κ*B pathway). Therefore, we use TNF-*α* to increase exogenous MUC4 in our study [17]. TNF-*α*-induced MUC4 was significantly decreased in both mRNA and protein level after being treated with ALA, and the effect was positively correlated with ALA concentration (Figure 3). In addition, we also proved that ALA inhibited gastric cancer cell invasion much strongly than anti-MUC4, which suggested that the inhibitory effect of ALA on gastric cancer was partly by suppression of MUC4 (Figure 4). These experiments demonstrated that ALA inhibited both endogenous MUC4 and MUC4 induced by TNF-*α* in gastric cancer cells, which may contribute to its inhibition on gastric cancer.

MUC4 is a downstream target gene of STAT3, and its expression is regulated by STAT3 [21]. In our study, both knockdown of STAT3 and treatment of ALA reduced MUC4 levels (Figure 5(a)), while overexpression of STAT3 reduced the effect of ALA on MUC4 (Figure 5(b)), and treatment of ALA did not change the level of STAT3 (Figure 5(c)). Therefore, we speculated that the mechanism of ALA inhibiting MUC4 was not to change the levels of upstream target gene STAT3, but to affect the function of STAT3.

STAT3 can be activated by various cytokines, including the interleukin 6 (IL-6) family of cytokines, granulocyte colony-stimulating factor, leptin, and epidermal growth factor [22]. STAT3 has redox-sensitive cysteines within its structure, and STAT3 is susceptible to redox regulation [23]. The cysteine thiol is very unstable and is readily oxidized to form disulfide bonds, which results in intermediate conformational changes within proteins [24]. Therefore, the change of redox state in the extracellular environment would affect the spatial conformation of STAT3 protein and further affect its function [25]. ALA can scavenge excessive ROS and regenerate endogenous antioxidants, which may influence the state of oxidative stress in cancer cells. Changes of redox in the cell are very likely to interfere STAT3 protein structure stability and affect its function. Consistent with our hypothesis, we proved that ALA blocked the binding of STAT3 to the MUC4 promoter (Figure 6). However, the mechanism by which ALA inhibits STAT3 binding to the promoter of MUC4 requires further extensive investigation.

## 5. Conclusion

In summary, ALA inhibits proliferation and invasion of gastric cancer cells through downregulation of MUC4. Specifically, ALA suppresses STAT3 binding to the promoter of MUC4. Our findings suggest that ALA could be used in the treatment of gastric cancer and even in the early stage of gastric cancer to inhibit the progress of gastric cancer.

## Figures and Tables

**Table 1:** The expression of MUC4 in the gastric cancer tissues and normal gastric tissues.

Groups	*n*	MUC4 expression		*p*
		Negative	Positive	
Gastric cancer tissues	240	56	184	<0.001
Normal gastric tissues	240	160	80	

**Table 2:** Correlations between MUC4 expression and clinic-pathologic factors.

Characteristics	*n*	MUC4 expression		*p*
		Negative	Positive	
Age				
≥65	112	9	103	<0.001
<65	128	47	81	
Gender				
Male	159	42	117	0.114
Female	81	14	67	
Tumor size				
≥5 cm	144	28	116	0.081
<5 cm	96	28	68	
Tumor location				
Upper	25	10	15	^†^0.103
Middle	87	29	58	^‡^0.144
Lower	128	31	97	^††^0.071
Depth of invasion				
T1+T2	102	36	66	<0.001
T3+T4	138	20	118	
TNM stage				
I+II	110	40	70	<0.001
III+IV	130	16	114	
Lymph node metastasis				
Present	134	20	114	0.001
Absent	106	36	70	
Distant metastasis				
Present	34	25	9	<0.001
Absent	206	31	175	

Note: ^†^upper *vs.* lower; ^‡^middle *vs.* lower; ^††^upper and middle *vs.* lower.

## Data Availability

The datasets used and analyzed during the current study are available from the corresponding author on reasonable request.

## Abbreviations

ALA: Alpha-lipoic acid
ROS: Reactive oxygen species
SOD: Superoxide dismutase
MUC4: Mucin 4
STAT3: Signal transducer and activator of transcription 3
FBS: Fetal bovine serum
qRT-PCR: Quantitative real-time PCR
ChIP: Chromatin immunoprecipitation.
